# 

**Published:** 1901-03

**Authors:** 


**This Journal and Atlanta Semi-weekly Journal one year for $1.00 cash in advance. No checks. Strictly to new subscribers.**

### NOTICE TO SUBSCRIBERS.

**Those of you who have not paid your subscription account will probably hear from a collection agency soon. We desire to explain that no offense is meant by our placing these accounts for collection. It is simply a matter of convenience. We cannot give the accounts as much attention as an agency can, hence are compelled to employ the agency.**

***NOTICE.—This JOURNAL and THE INTERNATIONAL JOURNAL OF SURGERY one year for $1.00 cash, to new subscribers. No out-of-town checks accepted unless 10c. is added for cost of cashing. Strictly to new subscribers.***

---

**This Journal and Atlanta Semi-weekly Journal one year for $1.00 cash in advance. No checks. Strictly to new subscribers.**

